# Langerhans Cells: Sensing the Environment in Health and Disease

**DOI:** 10.3389/fimmu.2018.00093

**Published:** 2018-02-01

**Authors:** Julie Deckers, Hamida Hammad, Esther Hoste

**Affiliations:** ^1^VIB Center for Inflammation Research, Ghent, Belgium; ^2^Department of Internal Medicine, Ghent University, Ghent, Belgium; ^3^Department of Biomedical Molecular Biology, Ghent University, Ghent, Belgium

**Keywords:** Langerhans cells, skin immunology, tumor microenvironment, atopic march, mouse models

## Abstract

In the last few decades, our understanding of Langerhans cells (LCs) has drastically changed based on novel findings regarding the developmental origin and biological functions of these epidermis-specific resident immune cells. It has become clear that LCs not only exert pivotal roles in immune surveillance and homeostasis but also impact on pathology by either inducing tolerance or mediating inflammation. Their unique capabilities to self-renew within the epidermis, while also being able to migrate to lymph nodes in order to present antigen, place LCs in a key position to sample the local environment and decide on the appropriate cutaneous immune response. Exciting new data distinguishing LCs from Langerin^+^ dermal dendritic cells (DCs) on a functional and ontogenic level reveal crucial roles for LCs in trauma and various skin pathologies, which will be thoroughly discussed here. However, despite rapid progress in the field, the exact role of LCs during immune responses has not been completely elucidated. This review focuses on what mouse models that have been developed in order to enable the study of murine LCs and other Langerin-expressing DCs have taught us about LC development and function.

## Introduction

Paul Langerhans first described Langerhans cells (LCs) in 1868. These cells were identified in the epidermis and reminiscent of neurons due to their dendritic morphology. Over a century later, Ralph Steinman discovered antigen presentation by dendritic cells (DCs) (1). LCs were subsequently classified as DCs (2) that are localized in the outermost layer of the skin, namely the epidermis, where they reside in close association with keratinocytes, the main epidermal cell type. LCs are also present in other stratified epithelia, such as the mucosal oral and vaginal epithelium. Recent studies show that these mucosal LCs functionally act as genuine LCs, although they originate from different precursors (3). In this review, we uniquely focus on LCs present in the skin epithelium. Keratinocytes undergo a specialized form of cell death, resulting in the formation of the cornified layers of the skin that are crucial in the establishment of a tight epidermal permeability barrier. Establishing a proper skin barrier is a prerequisite for terrestrial life, as it protects the body from dehydration and invasion of pathogens. Besides structural cells such as keratinocytes, the epidermis harbors different immune cell types, namely LCs, tissue-resident T-cells, and γδ T-cells (dendritic epidermal T cells; only in mice) that constitute the immunological skin barrier. Both keratinocytes and immune cells are essential for sensing the environment and function as a first line of defense against external insults (4). As such, LCs have proven crucial in various antimicrobial responses, which is reviewed in detail by West et al. in this research topic (5). For long, LCs were considered as the exclusive antigen presenting cells in the skin and a body of literature attributed a role to LCs in the pathogenesis of various skin diseases. However, other DC subtypes reside in the dermis and a plethora of recent evidence challenged the paradigm that centers LCs as the sole cell type responsible for T-cell priming to antigens in the skin. LCs differ ontogenically from dermal DCs, as LCs share a common precursor with macrophages, while dermal DCs are more closely related to conventional DC (cDC) subsets present in lymphoid tissues (6). Functionally, there appears to be a high level of redundancy between LCs and dermal DCs, whose specific phenotypes and functions are reviewed elsewhere (7, 8). LCs are able to migrate to the skin-draining lymph node, which is required for classical T-cell priming. LCs have recently been implicated in local immunosuppressive cutaneous reactions and activation of skin-resident memory T-cells, indicating an important role for LCs in mediating the adaptive phase of skin immunity (9, 10).

**Figure 1:**
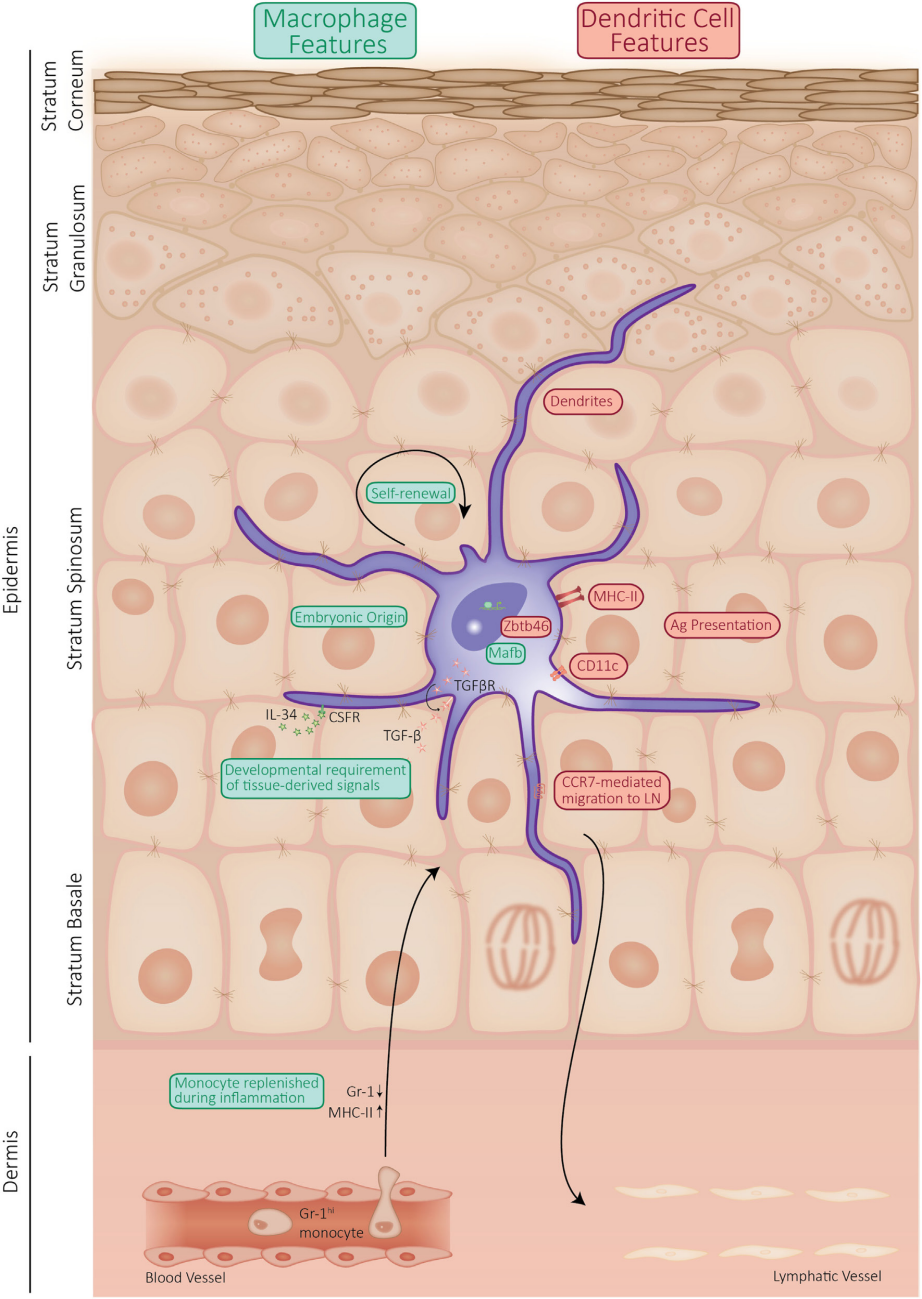
Schematic representation of the properties of Langerhans cell (LC) shared by dendritic cells (DCs) or macrophages. LCs display a mixture of properties, which they share with macrophages (indicated in green), such as self-maintenance and ontogeny. However, LCs are capable of presenting antigen and actively migrate to the draining lymph nodes, which qualifies them as DCs (properties shared between LCs and DCs are indicated in red).

Additionally, unlike cDCs, LCs have an embryonic origin and are largely maintained by self-renewal. In the past few years, there has been a lot of debate about the classification of LCs. Using developmental origin as a categorizing factor, which is encouraged in the complex field of the mononuclear phagocyte system (11), it is reasonable to classify LCs in the macrophage lineage. Expression of the transcription factor Zbtb46 enforces cDC identity (12). Recently, a highly elegant lineage tracing study corroborated the dual identity of LCs as these authors show that LCs express Zbtb46, while they originate from a *Mafb*-expressing progenitor, indicating macrophage origin (6). MafB was shown to control a network of self-renewal genes in proliferating resident macrophages (13). Like tissue-resident macrophages, LCs proliferate in a differentiated state, and these findings imply that MafB is likely involved in the proliferation of LCs. However, the specific molecular signals driving *in situ* LC proliferation remain to be elucidated. These findings nicely summarize the current persuasion of LCs being macrophages with DC functions. Phenotypically, LCs share many features with cDCs, whereas their ontology relates them to tissue-resident macrophages. Together, this shows that LCs represent a highly unique cell type, sharing features with DCs but arising from a different origin and exerting unique functions that distinguish them from cDCs, as summarized in Figure 1.

## Phenotype and Ontogeny

Langerhans cells make up 3–5% of all nucleated cells in the epidermis and occupy the *stratum spinosum*, a suprabasal epidermal layer that is characterized by “spiny” keratinocytes that undergo reinforcement of their cytoskeleton. LCs are stellate cells that protrude their dendrites *via* tight junctions toward the *stratum corneum* and as such can probe for antigens across several layers of the epidermis without disrupting the permeability barrier. LCs acquire a typical DC phenotype after birth by expressing major histocompatibility complex II (MHCII) molecules and the integrin αX chain (CD11c). Unlike mouse LCs, human LCs express low levels of CD11c and no F4/80, whereas they express CD1a and CD1c, two MHCI-related molecules involved in the presentation of lipid antigens (7). Epithelial cell adhesion molecule (EpCAM) regulates LC adhesion to keratinocytes and promotes LC migration to the draining lymph nodes (14). LCs express the C-type lectin Langerin (CD207), which is involved in the formation of Birbeck granules, endosomal organelles that are typical for LCs. These Birbeck granules have been shown to internalize viruses (15) and are thought to be part of the endosomal recycling complex (16). The presence of Langerin alone is not reliable to unambiguously define LCs, as Langerin expression has also been demonstrated on cDCs in the dermis, lungs, and spleen. Likewise, human and mouse LCs express SIRPα (CD172α), CD11b and CX3CR1, which is also expressed by most type 2 cDCs. Since the aforementioned markers are shared by different subsets of myeloid cells, multicolor flow cytometry with a minimal set of lineage imprinted markers is required for unequivocal identification of LCs in the skin and skin-draining lymph nodes (17).

Langerhans cells clearly share similarities with both DCs and macrophages (Figure 1). Doebel et al. recently summarized the emerging evidence of the dual identity of LCs, as they arise from macrophage precursors and acquire the unique properties of DCs in the epidermis (18). Indeed, LCs are radio-resistant cells from embryonic origin, which are maintained by self-renewal. With an estimated half-life of 2 months (19), LCs exhibit a slow proliferation rate in homeostatic conditions to replace dying and emigrating cells. In that aspect, LCs rather resemble tissue-resident macrophages than classical DCs, which are derived from bone-marrow precursors and depend on circulating progenitors to retain their high turnover rate (18, 20). Unlike cDC precursors that require FMS-like tyrosine kinase-3 (Flt3) signaling to differentiate, LCs develop independently of Flt3 and Fltl3 ligand (Flt3L). Of note, migrating LCs upregulate *Flt3* expression, suggesting a function of Flt3L in the activation of LCs (21). Like tissue-resident macrophages, LCs proliferate in a differentiated state and therefore express a set of self-renewal genes. However, the specific molecular signals driving *in situ* LC proliferation remain to be elucidated.

Even though LCs share ontogenic similarities with tissue-resident macrophages, they are, unlike macrophages, able to migrate to draining lymph nodes. LCs can take up and process foreign antigens in steady-state conditions and inflammation, which they can present to cells of the adaptive immune system in the lymph nodes. The migration of LCs is a multistep process involving a plethora of changes. First, LCs need to weaken their intercellular connections with the surrounding keratinocytes, which is partly regulated by E-cadherin (2). The release of E-cadherin provokes translocation of β-catenin, which is involved in the tolerogenic phenotype of DCs (22). Canonical Wnt signaling, which is mediated by β-catenin, is a master regulator of keratinocyte behavior in skin homeostasis and disease (23). It would, therefore, be interesting to investigate LC behavior in mouse models of aberrant cutaneous β-catenin activation in order to assess the involvement of LCs in altering keratinocyte behavior. In a next step, LCs need to cross the basement membrane in order to migrate into the dermis to transit to the lymph node. Therefore, they locally secrete collagen-degrading matrix metalloproteinases, enabling dermal rearrangements and transdermal LC motility (24). Once in the dermis, LCs can enter the lymphatics in order to travel to regional lymph nodes. Reseeding of LCs from the bone marrow into the epidermis occurs through strictly mediated routes along the hair follicle. Keratinocytes present in the region above the hair bulge secrete chemokine (C–C motif) ligand (CCL)-2 and CCL-20 (25).

A growth factor crucial for proper regulation of LC biology has proven to be transforming growth factor (TGF)-β and its associated transcription factors PU.1, Id2, and RUNX3. Mice deficient for one of these proteins lack fully developed LCs (26–28). TGF-β also drives differentiation of human precursor cells to cells with LCs characteristics *in vitro* (29, 30). TGF-β signaling can be induced both by keratinocytes and LCs. However, the finding that mice with a conditional deletion of TGF-β in LCs exhibits reduced LC numbers is indicative for a crucial autocrine loop for TGF-β in LC biology (31). PU.1 plays a pivotal role during many aspects of early hematopoiesis and myeloid cell differentiation and regulates the expression of the essential LC gene *Runx3* in response to TGF-β (32). Interestingly, LCs under steady-state and inflammatory conditions exhibit differential requirements for the TGF-β-induced transcription factor ID2, supporting the finding that inflammatory LCs arise from different precursors than their steady-state counterparts (32, 33). The late endosomal adaptor molecule p14 (LAMTOR2) is also indispensable for LC homeostasis and this appeared to be due to changes in TGF-β sensitivity (34, 35). Interestingly, p14-deficient LCs exhibit an increased sensitivity to apoptotic stimuli and defective proliferation, partially due to downregulation of the TGF-β receptor II. Another member of the TGF-β family shown to be important for the early steps of LC differentiation is bone morphogenetic protein (BMP) 7. Mice lacking BMP7 have significantly reduced LC numbers, and LCs appear less dendritic than in control littermates (36). These authors showed that the development of human LC could be driven by BMP7 in the case of redundant TGF-β signaling, as is the case in prenatal epidermis and in the basal epidermal layer (36). In conclusion, the number of LCs in the epidermis in homeostatic conditions is maintained by self-renewal that replenishes the constant low-level migration from the epidermis to the draining lymph nodes. The development, differentiation, and proliferation of LCs are tightly controlled by a network of transcription factors, cytokines, and growth factors, which are highly influenced by the tissue microenvironment.

## LCs in Cutaneous Inflammation and Wound Healing

Langerhans cells are, due to their location within stratified epithelia, part of the first line of defense to pathogens present in the environment. Non-activated LCs are constantly migrating to the lymph nodes to present self-antigen and establish immune tolerance in homeostatic conditions (37, 38). During pathogenic encounter, germ line-encoded pathogen-recognition receptors (PRRs) recognize molecular patterns on microbes, resulting in the secretion of pro-inflammatory cytokines and subsequent enhanced mobilization of LCs to the lymph nodes (39). The functional importance of LCs in infection and their interaction with PRRs is extensively reviewed by West et al. in this research topic (5).

Cutaneous inflammation, infection, or injury provokes extensive migration of LCs from the epidermis, generating an empty niche that is repopulated by an initial wave of short-lived LCs arising from circulating Gr-1^hi^ monocytes (33, 40). Seré et al. reported that these monocyte-derived LCs that infiltrate the skin during inflammation are short-lived and independent of Id2, but their recruitment into the skin depends on CCR-2 and -6 (33). These short-lived LCs are gradually replaced by a second wave of Id2-expressing LCs that remain long-term in the epidermis and express high levels of Langerin, CD24, and EpCAM, which distinguishes them from short-lived LCs (33). These observations indicate the importance of distinct mechanisms for LC development in homeostatic versus inflammatory conditions. LCs are imprinted by the epidermis as they require keratinocyte-derived interleukin (IL)-34 for their development, homeostasis, and regeneration (41).

Wound healing responses in the skin involve multiple cellular rearrangements to ensure rapid restoration of epidermal permeability barrier function. Regenerative cutaneous responses can be subdivided into multiple phases, namely the inflammatory, new tissue formation, and tissue remodeling stage that are each characterized by specific molecular and cellular responses (42). Keratinocytes that are stressed during wounding rapidly upregulate ligands for the lymphocyte activation receptor natural killer group 2D (NKG2D), resulting in migration of LC populations out of the epidermis, followed by the appearance of αβ T-cells in the epidermis (43). Enhanced expression levels of the murine NKG2D ligand Rae-1ε have been observed in wounded skin, and mice lacking NKG2D exhibit delayed cutaneous wound healing (44), corroborating the importance of the cross talk between keratinocytes and LCs during regenerative processes in the skin. This intercellular interplay is already apparent in response to minor injury, such as repetitive tape-stripping of the epidermis (45). In these inflammatory conditions, LCs have been shown to penetrate the tight junctions that link keratinocytes, enabling endocytosis of antigens (46). LC–keratinocyte cross talk is in part established by the production of pro-inflammatory cytokines, such as IL-1, granulocyte macrophage colony stimulating factor (GM-CSF), and tumor necrosis factor (TNF), which are stimulators of LC migration (47). Hitherto, it is not known whether those cytokines can directly alter LC behavior *in vivo* or whether this occurs indirectly *via* keratinocyte activation. Repopulation of the epidermis by LCs occurs in the final stages of wound healing, namely the tissue-remodeling stage (48). It remains unclear whether LC repopulation after wounding occurs through substantial infiltration of monocyte-derived LCs or through an initial wave of a small population of infiltrating LCs cells that proliferate *in situ*. *In vitro* data using primary human dermal lymphatic endothelial cells show that stimulation with TNF results in overexpression of key surface adhesion molecules (49). In human non-ulcerated epidermis from diabetic patients, which have markedly delayed cutaneous reparative responses, an increase in LCs was observed relative to normoglycemic patients. Higher numbers of LCs present in diabetic foot ulcers were associated with a better healing outcome, pointing to a beneficial role for human LCs in cutaneous reparative inflammation (50). In contrast, the observation that injury repair of the skin occurs normally in mice lacking the PU.1 transcription factor, which is crucial for LC development (32), would argue for a redundant role of LCs in cutaneous regenerative responses (51). Clearly, the role of LCs in mediating the different stages of wound regeneration is still under debate, and it remains to be investigated whether targeting this cell population would be an interesting therapeutic avenue in managing wound-induced acute or chronic inflammation.

## LCs in Allergic Skin Reactions

Langerhans cells have been widely studied in allergic skin reactions. However, the role of LCs in contact hypersensitivity and allergic sensitization remains controversial.

### Contact Hypersensitivity

Allergic contact dermatitis occurs in individuals that mount a type IV delayed-type hypersensitivity against contact allergens, mostly small organic molecules with chemical reactivity (so-called chemical sensitizers such as metals, topical antibiotics, or preservatives). These chemical sensitizers penetrate the cornified skin layers due to their small size and covalently bind to epidermal proteins to form the so-called “neo-antigens” that can be recognized by APCs (52). This process of haptenization can be bypassed experimentally by applying haptens to murine skin. The subsequent tissue responses mimic those arising during allergic contact dermatitis in humans. LCs are one of the first APCs to encounter these haptens but their exact role in CHS remains unclear due to conflicting results obtained from different mouse models, which is extensively reviewed elsewhere (53).

Initial reports demonstrated that depletion of LCs could not completely abrogate CHS responses to haptens (54, 55). This emphasized the redundancy of different DC subsets in the epidermis and dermis, since dermal DCs can also be activated by haptens as these are small enough to penetrate into the dermis. Studies aiming to unravel the role of LCs in CHS made use of different mouse models to delete functional LCs, which resulted in contradictory findings. Genetic ablation of LCs can be achieved by injecting diphtheria toxin in mice expressing the human or simian diphtheria toxin receptor (DTR) driven by the *CD207* (Langerin) promoter. This toxin blocks protein translation and induces cell death only in cells that transgenically express DTR, as the mouse ortholog of this receptor is significantly less sensitive to the toxin (56). In the skin of Langerin-DTR mice, all LCs and Langerin^+^ cDC1s can be depleted by a single injection of DT. By using this transgenic system, two reports showed that LCs were largely dispensable for the induction of CHS (54, 55). However, the notion that dermal Langerin^+^ cDC1s are also depleted in these mice calls for reconsideration of previous findings. In order to specifically investigate the contribution of LCs in CHS, CHS responses can be assessed 1 week after DT injection, since Langerin^+^ cDC1s are replenished from circulating precursors within 7 days, whereas LCs remain absent from the epidermis for at least 2 weeks. T-cell priming to topically applied haptens was diminished once Langerin^+^ cDC1s were reconstituted, while LCs were still depleted, indicating a role for LCs in CHS sensitization (57). Nevertheless, a functional redundancy exists between LCs and Langerin^+^ cDC1s, depending on the hapten dose (58).

An alternative method to assess the importance of LCs in CHS responses is to make use of human (h) Langerin-DTR mice, which constitutively lack LCs in the presence of functional Langerin^+^ cDC1s due to a different promoter regulation of the human *CD207* gene. Surprisingly, these mice showed an enhanced cutaneous sensitization to haptens, suggesting a suppressive role of LCs in CHS (59). However, these conclusions were refuted by other reports demonstrating that CHS responses were reduced in mice that only lack LCs due to conditional deletion of TGFβ receptor 1 or p14 in the Langerin^+^ cells (35, 60). Indeed, LCs have been implicated in the induction of regulatory T-cells and are crucial for establishing tolerance to mild contact allergen sensitization by expanding regulatory T-cells while deleting allergen-specific CD8+ T-cell responses (61–63). Accordingly, transgenic overexpression of RANKL by keratinocytes reduces CHS responses, and this was shown to be mediated by an enhanced capacity of LCs to expand CD4^+^CD25^+^ regulatory T-cells (64). These suppressive functions of steady-state LCs were reported earlier in human skin, where LCs were shown to activate skin-resident regulatory T-cells (10). Interestingly, these studies demonstrated that the activation state of LCs determined their immunological response, as activated LCs induced effector T-cells and limited regulatory T-cell activation both in mice (63) and humans (10). In conclusion, by studying CHS responses to haptens, it became clear that LCs can contribute to contact allergen sensitization depending on the hapten dose and on the experimental mouse model that is used.

By making use of a transgenic mouse model expressing CD1a, a lipid-presenting protein normally not present in mice, it was shown that LCs that transgenically expressed CD1a showed a suppressed CHS to dinitrofluorobenzene. However, these CD1a^+^ LCs were able to induce Th17 responses to the topical application of inflammatory plant lipids (65). This indicates that the nature of the hapten can provoke antigen presentation by LCs that skews T-cell responses in a specific direction. Nevertheless, it remains unclear why in some patients tolerance is broken and contact dermatitis occurs, and how LCs are involved in this process. In conclusion, it is crucial to take into account the dose, nature, and application route of the hapten when drawing conclusions from mouse models of CHS. Also, the loss of LCs due to a severe inflammatory stimulus or injury will be replenished by circulating Gr-1^hi^ monocytes, whereas the loss of LCs due to a less severe stimulus can be resolved by local proliferation of LCs (20). This implies again that the composition of the LCs pool and their contribution to CHS may vary between experimental models.

### Allergic Sensitization to Protein Antigens

Like contact allergens, protein allergens from, e.g., mold, pollen, house dust mite (HDM), and cockroach can be present in high amounts at the skin surface. However, when studying allergic sensitization, it is important to note that haptens easily penetrate into the deeper skin layers and activate dermal DCs, whereas it is until now unclear whether protein allergens are able to do so without prior barrier disruption. Therefore, many experimental models inflict epidermal barrier disruption prior to protein antigen exposure, for instance by tape stripping or by using irritants such as acetone. These handlings will also activate keratinocytes and immune cells in the skin and induce mild inflammation (66). Consequently, epidermal barrier perturbation, prior to allergen exposure, influences the outcome of diverse experimental models used to study allergic responses and should be taken into consideration. Nakajima et al. reported that LCs are crucial for epicutaneous sensitization to ovalbumin (OVA) (67), whereas we recently demonstrated that dermal type 2 cDCs and not LCs are responsible for skin sensitization to HDM (68). We found that LC-depleted mice can be efficiently sensitized to HDM *via* the skin and HDM-loaded LCs are unable to transfer sensitization to naïve mice (68). The major discrepancy between these models lies in the fact that OVA is an inert protein that requires prior tape stripping, which enables penetration and functions as an adjuvant by activating keratinocytes and immune cells, whereas the HDM extract itself contains proteolytic active enzymes and contaminating endotoxins that can activate keratinocytes and immune cells to produce pro-allergic cytokines. The fact that these models induce keratinocyte activation in a different manner could explain divergent roles for LCs during sensitization. Emerging evidence reveals an important interplay between epithelial cells and DCs during allergic sensitization, both in the lungs and in the skin (69). We previously showed that endotoxin-induced toll-like receptor (TLR4) signaling on bronchial epithelial cells is required for Th2 priming to HDM (70) *via* production of Th2-skewing cytokines (71). Likewise, activated keratinocytes can produce typical pro-allergic cytokines that skew DCs to prime Th2 cells, such as thymic stromal lymphopoietin (TSLP), IL-33, IL-1α, and GM-CSF (4). Interestingly, TSLP stimulates human LCs to induce pro-allergic T-cells (72). Of note, the pro-allergic maturation effect of TSLP was restricted to epidermal LCs and does not occur in CD34^+^-derived LCs (73). However, LCs are dispensable for the TSLP-driven Th2 priming in a mouse model where TSLP overproduction is evoked by application of a vitamin D3 analog (74). Remarkably, we found that neither barrier perturbation prior to sensitization nor proteolytic activity present in HDM is required for efficient epicutaneous sensitization to HDM (68).

Although we did not find a crucial role for LCs during skin sensitization to HDM, we demonstrated that the lack of LCs results in increased production of type 2 cytokines by HDM-restimulated T-cells in the lymph nodes (68). Our findings are in line with previous studies showing that LCs can exert regulatory functions (9, 10, 62) and thus might also be able to suppress Th2 priming to HDM. Again, as in CHS, these contradictory findings indicate that multiple factors determine whether and how LCs contribute to epicutaneous sensitization. For instance, if allergens get in contact with the disrupted skin barrier of genetically predisposed individuals, they may circumvent the potential suppressive homeostatic function of LCs and gain direct access to dermal DCs. Also, in patients with a minimal degree of inflammation in the skin, LCs might be skewed toward a pro-allergic state. In conclusion, the exact role of LCs in epicutaneous sensitization is yet to be elucidated, and recent findings point to a high functional redundancy between different skin DC subsets.

## LCs in Atopic Dermatitis (AD)

**Figure 2:**
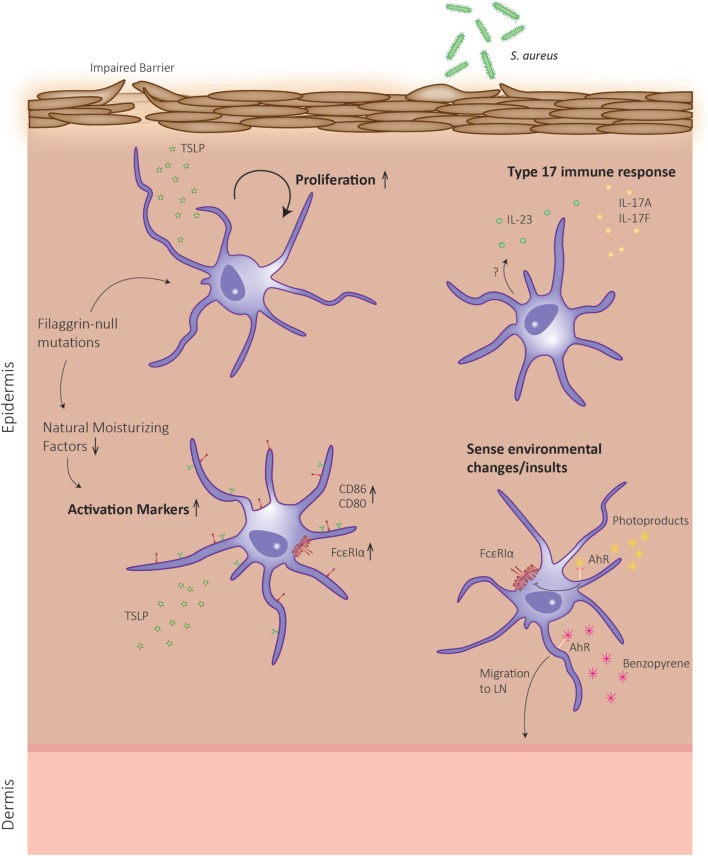
Schematic representation of Langerhans cell (LC) functions in atopic dermatitis (AD). In skin of AD patients, a defective barrier can be due to mutations in the gene encoding for the structural protein filaggrin. During keratinocyte differentiation, the filaggrin monomer is degraded to the level of natural moisturizing factors, which can alter dendritic cell behavior. The defective epidermal barrier leads to the induction of LCs, which can enhance their proliferation rates and exhibit an enhanced activation state. Upon sensing environmental insults, an additional type 17 response is mounted, and LCs migrate to the lymph node to present antigen.

Eczema or AD is a Th2-driven disease that often precedes allergic or asthmatic responses in a progressive process termed the atopic march. Even though the role of LCs in sensitization to protein allergens is still under debate, LCs might also contribute to the effector phase of allergic reactions in the skin, causing symptoms of AD (Figure 2). However, reports from AD patients are limited and the exact role of LCs in this setting remains to be elucidated. In skin biopsies from AD patients, more LCs are present, although it is unclear whether these are skin-resident or monocyte-derived LCs (75). These authors show that TSLP causes proliferation of skin-resident LCs, while unaffecting the proliferation of a marginal wave of monocyte-derived LCs (75). Human LCs are in an activated state in AD lesions as demonstrated by enhanced CD80 and CD86 expression (76), which was corroborated by the observation that murine LCs also express increased maturation markers in a mouse model of AD and this correlated with AD severity (77).

Human skin with defective epidermal barrier function, as is the case in patients with mutations in the *filaggrin* gene encoding for a major structural protein in keratinocytes, predisposes to AD development (78). Interestingly, it was recently shown that in skin biopsies of patients harboring filaggrin-null mutations, LCs are in a more activated state compared to control patients, irrespective of whether these patients suffered from AD (79). These authors demonstrate that filaggrin breakdown products, which act as natural moisturizing factors in the skin, reduce the maturation phenotype of *ex vivo* generated monocyte-derived DCs. Together, this indicates that filaggrin mutations can affect LC function (Figure 2), but whether this results in LC hyperactivation that instigates AD symptoms remains to be explored.

Another indication that LCs might exert an important role in the effector phase of AD is the observation that LCs from AD lesions display increased expression levels of the high-affinity immunoglobulin (Ig)E receptor (FcεRI) (80, 81). This receptor binds allergen-specific IgE molecules, thereby promoting antigen focusing to the cell surface and subsequent antigen uptake (82). Interestingly, in human CD34^+^-derived LCs, TLR2 ligation by *Staphylococcus aureus* products resulted in downregulation of FcεRI expression (83).

Recently, it was postulated that the AD-resolving effect of phototherapy could be mediated by LCs. Ultraviolet (UV)B irradiation generates photoproducts that activate the aryl hydrocarbon receptor (84), which in turn downregulates FcεRI in *ex vivo* generated, CD34^+^-derived LCs (85). Additionally, a recent report showed that activation of AhR by benzopyrene present in cigarette smoke induced LC mobilization in association with reduced E-cadherin levels in keratinocytes (86). Moreover, AhR-deficient LCs showed an impaired maturation, which resulted in a reduced CHS to haptens (87). Both events indicate that benzopyrene could either directly or indirectly activate pro-inflammatory pathways in LCs that may worsen AD, but this remains to be investigated. AhR expression levels are also increased in AD skin (86), and this receptor was recently proposed to link air pollution to AD (88). Together, these reports imply that the nature of AhR ligand could define a pro- or anti-inflammatory stimulus for LCs that may contribute to disease outcome.

Langerhans cells are important in raising cutaneous Th17 responses to certain bacterial infections. Interestingly, HDM-specific T-cells from AD patients were found to present either a Th2, Th17, or Th2/Th17 phenotype (89). Moreover, the percentage of Th17 cells and the level of circulating IL-17/IL-23 cytokines in peripheral blood of AD patients correlate with disease severity (90, 91). How exactly this additional Th17 response arises in AD, a typical Th2-mediated disease, remains unclear. One possible explanation is that a defective barrier, induced by genetic defects or scratching, could facilitate microbial dysbiosis of the skin. Indeed, AD patients regularly suffer from concomitant *S. aureus* colonization, which aggravates the disease (92). Kobayashi et al. demonstrated that mice that develop eczematous skin with a disrupted epidermal barrier are more prone to develop *S. aureus* colonization and concomitant cutaneous Th17 immune responses. This inflammatory phenotype was mediated by infiltrating γδ T-cells and Th17 cells and was abrogated when mice lacked LCs (93). Likewise, human LCs were shown to have a high capacity to induce IL-22 production by γδ T-cells, which in turn have potent clearing effects on *S. aureus* (94). In conclusion, LCs can exert different functions during AD depending on the stimuli they receive from the microenvironment. This explains why, despite many efforts, researchers have hitherto been unable to ascribe an unambiguous role for LCs in AD.

## LCs in Psoriasis

Psoriasis is a chronic, inflammatory skin disorder manifested by the presence of silvery scaling on the affected regions and characterized by increased proliferation and abnormal differentiation of keratinocytes. In psoriatic skin, there is an accumulation of immune cells, of which DCs and T-cells are the most essential (95). The current knowledge on the role of LCs in human psoriatic patients is reviewed extensively in another review article in this research topic (5). In mice, a psoriasis-like immune response in the skin can be mimicked by topical application of the TLR7 and -8 ligand imiquimod (IMQ), and the dermatitis induced in this experimental model depends on IL-23/Th17 signaling (96). LC-depleted mice show decreased IMQ-induced dermatitis and significantly reduced levels of IL-23, indicating that LCs are a major source of IL-23 in this model (97). In contrast, Wohn et al. reported that cDCs, and not LCs, are responsible for IL-23 production and subsequent dermatitis (98). These contradictory findings, both deduced by using the same Langerin-DTR mouse strain, might be due to the difference in topical IMQ application, namely on the ear versus the back skin. This could indicate that based on their location within the body, LCs might induce different tissue responses.

Mice that ectopically express the human lipid-presenting protein CD1a on LCs exhibit an aggravated psoriatic response on IMQ treatment, which could be reduced by administration of a CD1a-blocking antibody. Also in human patients, the inflammatory cytokine response typical for psoriasis was blocked by treatment with anti-CD1a antibodies (65). These observations exemplify the importance of lipid antigen presentation in the pathogenesis of psoriasis. Among the inflammatory mediators of psoriasis, TNF and IL-1β have been proven to be essential components of the cytokine storm that is typical in active flares of the disease (99). Recently, it was demonstrated that the main DCs responsible for the production of these pro-inflammatory cytokines are monocyte-derived DCs, including a population of LCs (100). These recent observations reemphasize the role of LCs in mounting the Th17 arm of the immune response that occurs in psoriatic skin. As neutralizing IL-17α is highly effective in the treatment of psoriasis (101), specific therapeutic targeting of LCs in the context of psoriasis might therefore represent an interesting therapeutic avenue.

## LC-Associated Pathologies

Langerhans cell histiocytosis is a disorder of unknown etiology with varying clinical manifestations and disease severity, which is characterized by the infiltration of activated DCs in multifocal lesions (102, 103). A common clinical feature of LC histiocytosis is a skin eruption soon after birth, which may resolve spontaneously or spread to other regions of the body. Although Birbeck granules have been observed in cells within these lesions, the LC origin of these histiocytoses has not been formally proven. LC histiocytoses encompass many subtypes with a wide range of clinical manifestations, which are characterized by inflammation and hyperproliferation of leukocytes and can involve skin, bone, lung, bone marrow, central nervous system, and lymph nodes (104). Recent reports have demonstrated that in two thirds of patients activating mutations in the RAF/MEK/ERK pathway occur within LC histiocytic lesions (105, 106). Current therapeutic treatments encompass MEK or BRAF inhibitors or a combination of both, as recently reviewed by Haroche et al. (104). The current mouse model developed to study LC histiocytosis, by transgenic expression of the simian virus 40-derived oncogenes under control of the CD11c promoter, not only mimics certain features of the disease but also exhibits hallmarks of LC sarcoma (107). Therefore, this animal model might not represent the best tool to study the etiology of LC histiocytosis.

Langerhans cell sarcoma is a rare neoplastic LC disorder that can form *de novo* or can arise from LC histiocytosis (108). So far, no recurrent chromosomal modifications have been identified in both disorders, but further studies to correctly classify and potentially stratify these diseases are necessary.

## LCs in Cancer

Non-melanoma skin tumors include basal cell carcinomas and squamous cell carcinomas (SCCs), which typically exhibit a severely reduced LC presence in the peritumoral and tumoral regions (109). As DCs are able to elicit potent antitumor immune responses, it is generally excepted that they play a crucial role in cancer immune surveillance. However, DCs can exhibit functional alterations in certain tumor types and become less potent in T-cell stimulation and production of interferons, enabling escape from immune surveillance (110, 111). Various reports point to a crucial role for LCs in mediating malignant progression in the skin. In human SCCs, suppression of DC functionality was observed, while LCs isolated from SCCs have been shown to be potent type 1 immune stimulators *in vitro* (112). Interestingly, LCs migrate abnormally from SCC skin and have reduced T cell priming abilities within the lymph node (113). Many cytokines that are known to mediate LC migration out of the dermis, such as TSLP, IL-1, and TNF, have been implicated in cutaneous cancer models (114–116). Aberrations in the epidermal permeability barrier prime the secretion of these pro-inflammatory cytokines, resulting in altered cancer susceptibility (117). However, it remains to be established whether in these conditions, enhanced LC migration is an underlying cause of skin tumorigenesis. Given the recent discovery of Langerin-expressing DCs beyond LCs, it is plausible that many of the tumor biology effects that were attributed to LCs are in fact due to other Langerin^+^ cells in the skin, and therefore, these observations need to be revisited. Human LCs have been shown to be more efficient activators of naïve CD8^+^ T-cells than dermal DCs, which might have important implications for antitumor responses (118). In the widely established two-stage DMBA/TPA model of chemical skin carcinogenesis, Langerin-deficient mice are fully protected from papillomagenesis and subsequent SCC formation (119). These authors demonstrate that Langerin^+^ cells can enhance DMBA-induced DNA damage, although in light of recent findings, it remains to be established whether the LCs are the main Langerin^+^ DCs that are capable of metabolizing DMBA. Indeed, various reports now show that the DMBA-metabolizing activity of LCs is dispensable for inflicting H-Ras mutations in keratinocytes (120, 121).

Treatment with ionizing radiation is frequently used as a localized tumoricidal therapy in cancer patients; however, recent data point to the induction of additional antitumor immune responses by radiotherapy, such as infiltration by regulatory T-cells within the tumor and activation of APCs (122, 123). LCs are remarkably radio-resistant and are highly potent in rapidly repairing the inflicted DNA damage, which was shown to be mediated by CDKN1A (cyclin-dependent kinase inhibitor) (124). Repetitive damage by UVB irradiation predisposes to melanoma formation and given the staggering success of immunotherapeutic interventions in melanoma patients, it is pivotal to understand the immune cell dynamics within the melanoma microenvironment. Based on the coexpression of S100 (125) and CD1, a reduction of LCs in the epidermis overlying melanoma has been reported (126). This reduced number of LCs might represent an early event after UVB photodamage, as LC migration to the lymph nodes occurs after UVB irradiation (19). A recent report demonstrates that keratinocytes reduce αvβ6 and αvβ8 integrin expression after UVB damage, resulting in downregulation of TGF-β signaling in LCs and subsequent migration of LCs out of the epidermis (127).

During malignant conversion of tumors, epithelial cells can lose their cell polarity and adhesive properties in order to become more invasive and mesenchymal. LCs might also play a role in these epithelial–mesenchymal transitions (EMT) in cutaneous cancers, as many molecules that are involved in mediating LC migration in and out of the epidermis are known to play a role in various stages of the EMT process (128). TGF-β as a major transcription factor involved in LC behavior also acts as a master regulator of EMT processes in skin cancers (129). BMP7 is important to maintain LCs in the epidermis in a rested state. In cancers, BMP7 plays a similar role as it promotes reversed EMT, namely mesenchymal to epithelial transition, clearly acting as a homing signal to epithelia (36, 130). One of the signature events that characterize EMT is the loss of E-cadherin expression, which as mentioned above is a prerequisite for LC migration out of the epidermis (2). Interestingly, as mentioned above, secreted E-cadherin induces β-catenin stabilization, an event that occurs in various cutaneous tumors and leads to tolerogenicity in DCs (22, 23). Whether tolerogenic LCs contribute to tumorigenesis in Wnt-activated cutaneous tumors, remains to be investigated. The receptor tyrosine kinase Met is expressed on all DCs and was originally characterized as an oncogene. Met signaling is crucial for the migration of LCs from the skin to the lymph nodes as LCs in mice that lack Met do not reach the skin-draining lymph node in inflammatory conditions (131). Interestingly, Met signaling mediates the enzymatic activity of MMP-2 and -9, the aforementioned proteases that are crucial for LCs to breach the basement membrane when emigrating out of the epidermis (132).

## Discussion and Outstanding Questions

Despite the fact that LCs were first described over a century ago, there are still many outstanding questions regarding their biological functions and redundancies in skin homeostasis and pathology. The identification of dermal subsets of Langerin^+^ DCs, independent of epidermal LCs that are in transit to skin-draining lymph nodes, questioned the validity of the LC paradigm and therefore the biological function of LCs in different pathological contexts needs to be revisited. In order to better understand LC biology, more extensive studies on human LC biology are needed. Highly elegant studies have recently indicated the complexity of human DCs, unveiling the existence of multiple pre-DC precursors that have distinct functional properties (133). There is also a need for mouse models enabling specific targeting of LCs, without affecting other DC subsets. The use of mouse models that enable inducible targeting of LCs might be beneficial over constitutive LC deletion, as it might well be that life-long absence of specific leukocyte subsets affects normal skin development and homeostasis. A better understanding of the relative contributions of skin-resident DC subtypes in mounting the plethora of tissue responses that occur in the skin upon various insults and trauma will improve the development of DC vaccines and other targeted therapies.

The skin hosts trillions of microorganisms that have been proven crucial for tissue homeostasis and immunity. It was shown that mice lacking functional LCs mount normal T-cell responses relative to control littermates following infection with the skin commensal *Staphylococcus epidermidis* (134). However, LCs are crucial in raising a humoral protective response after topical application of the *S. aureus*-derived exfoliative toxin A (135). These data point to the potential use of Langerin-based therapies in protection from infection, although microbial restriction by LCs seems to be based on receptor usage and hence can only be deployed against certain microbes (136). Whether LCs are key players in establishing a healthy skin microbiome remains to be investigated. It would also be worthwhile to investigate whether LCs develop normally in the absence of commensal microbes as would be the case in germ-free animals. It is clear that altering LC functions could be an interesting therapeutic avenue in various disease contexts. Glycans are crucial determinants in the binding of C-type lectin receptors, such as Langerin, to their ligands and can be modified in order to alter immune modulation on many levels, from immune recognition of pathogens to regulating adaptive immune responses, which could be exploited to modulate immune surveillance, as reviewed elsewhere (137, 138). In the current era of single-cell technologies, it is possible to question whether multiple subsets exist within the LC population and how these could affect tissue immune responses. Such studies might shed light on the controversial roles that have been attributed to LCs in infection and cancer and would pave the way for therapeutic targeting of this unique cell-type.

## Author Contributions

JD and EH drafted and revised the manuscript. HH revised the manuscript.

## Conflict of Interest Statement

The authors declare that the research was conducted in the absence of any commercial or financial relationships that could be construed as a potential conflict of interest.
